# Synthesis of Diblock Polyampholyte PAMPS-b-PMAPTAC and Its Adsorption on Bentonite

**DOI:** 10.3390/polym11010049

**Published:** 2018-12-30

**Authors:** Ling Lin, Yuanhao Luo, Xin Li

**Affiliations:** 1 School of Chemistry and Chemical Engineering, Southwest Petroleum University, Chengdu 610500, China; 2 Oil & Gas Field Applied Chemistry Key Laboratory of Sichuan Province (Southwest Petroleum University), Chengdu 610500, China; 3 CCDC Drilling Fluid Technology Service Company, Chengdu 610051, China; haolyeureka@163.com; 4 Petroleum Engineering School, Southwest Petroleum University, Chengdu 610500, China; up96157@gmail.com

**Keywords:** block polyampholyte, bentonite, adsorption, AMPS, MAPTAC

## Abstract

To study the adsorption of polyampholyte on bentonite (Bent), a block polyampholyte, PAMPS-b-PMAPTAC, comprised of 2-Acrylamido-2-Methylpropane Sulfonic Acid (AMPS) units and Methacrylamido Propyl Trimethyl Ammonium Chloride (MAPTAC) units, was synthesized using reversible addition-fragmentation chain transfer polymerization (RAFT) method. The block polyampholyte samples were characterized by FTIR, ^1^H NMR and Gel Permeation Chromatography (GPC). The microstructure of block polyampholyte and random polyampholyte in deionized water indicated that uneven distribution of charged groups increased the entanglement of polymer chains. Addition of salt weakened the electrostatic interactions among charged groups, and, therefore, increased the zeta potential of polyampholyte in aqueous solutions. The adsorptive behaviors of PAMPS-b-PMAPTAC on Bent were studied using elemental analysis, and the effects of external factors were considered. The adsorption equilibrium of polymers on Bent was reached after 12 h. Increased temperature and increased salinity exerted a positive and negative effect on the adsorption of polyampholyte, respectively. The molecular weight played as the decisive factor for the adsorption of polyampholyte in the absence of NaCl, while the content of cationic groups acted as the main factor in the presence of NaCl. Block polyampholyte exhibited higher adsorption than random polyampholyte in the absence of salt. XRD results also indicated that block polyampholyte had a better intercalation effect than random polyampholyte.

## 1. Introduction

Polyampholyte is one kind of polymers with both cationic groups and anionic groups distributed on the same backbone. In aqueous solutions, these two kinds of charged groups in polyampholyte deionize, and the chains carry opposite charges. Driven by the electrostatic interaction and Van der Waal’s force, polyampholyte chains assume various conformations, namely pole regime, pancake regime, and fence regime, under different conditions [1]. The conformation and behavior of the polymers in aqueous solutions, therefore, rely primarily on the monomer nature, charge asymmetry, charge distribution and chain length.

In terms of the monomer unit distribution in the backbone, polyampholyte can be classified as random polyampholyte with charged groups distributed statistically along the chain, alternating polyampholyte, and block polyampholyte with ionized monomer units bearing like charge located in different regions. In the laboratory, random polyampholyte is often synthesized by two methods. The first method is to add cationic monomers and anionic monomers, sometimes with other monomers, into one flask containing an aqueous solvent, and then initiate the polymerization. A number of reported random amphoteric polymers were produced in this one-pot approach [2,3]. The second method is to synthesize a cationic copolymer comprising cationic moieties and allyl units, followed by a hydrolyzation using NaOH or HCl to transform the allyl units to negatively-charged carboxyl units [3,4,5]. Using the two-step approach, You et al. obtained a cellulose-based polyampholyte by introducing both quaternized ammonium groups and a block of acrylic acid units via grafting [6].

Block polyampholyte is a class of responsive polymer attracting growing attention. The architecture of block polyampholyte includes AB diblocks [7,8], ABA triblocks [5,9,10,11], and other multi-blocks [12]. In the presence of weak acidic groups or weak basic groups along the backbone, block polyampholyte can change the net charge sign between positive and negative upon pH variation. Rich behaviors of block polyampholyte can be observed when external stimuli such as pH variation and temperature change is triggered. The dynamic protonation–deprotonation equilibrium of weak acid blocks (e.g., AA, stands for acrylic acid) and tertiary ammonium moieties (e.g., P2VP, stands for poly(2-vinyl-pyridine)) upon pH change endows the block polyampholyte with pH-responsive behavior. Such behaviors can be found in the presence of polyacrylic acid (PAA) block and tertiary ammonium group block [7], or PAA block and P2VP block [13,14]. In these cases, at low pH, both PAA block and P2VP block were protonated, and only the later was ionized and the polymer chain exhibited the same behavior as polycation; at intermediate pH, deprotonated AA groups and protonated 2VP groups attracted each other. If the charge asymmetry was not significant, e.g. equimolar addition of anionic and cationic groups, the polymer chain tended to collapse into globular conformation and precipitate from the solution, as a result of intensified attractions between oppositely charged moieties [10]. When pH increased to over 8, both AA groups and 2VP groups underwent a process of deprotonation, and the polymer chain carried net negative charges and exhibited a behavior of polyanion. Dyakonova et al. argued that the contribution of electrostatic interactions includes Coulombic attraction and entropy gain through counterions release [10]. If hydrophobic association is introduced along with Coulombic interaction into the inter- and intra- chain interaction, polyampholyte may form a pH-responsive and temperature-responsive hydrogel [5,15].

Both random polyampholyte and block polyampholyte were applied in the area of drug delivery, tissue engineering, membrane, water treatment, and so on [16,17]. Mishra et al. delivered indomethacin using a random polyampholyte, poly(methacrylamido propyl trimethyl ammonium chloride/methacrylic acid), where the polyampholyte increased the release of indomethacin [18]. A random polyampholyte hydrogel consisting of [2-(methacryloyloxy)ethyl]-trimethylammonium chloride and 3-sulfopropyl methacrylate potassium salt had adjustable mechanical properties without affecting the non-fouling properties [19]. A membrane containing both sulfonic groups and quaternary ammonium groups exhibited a high selective separation of bovine serum albumin and lysozyme [20]. Copello et al. reported a random polyampholyte bearing carboxylate and 2-methylimidazole groups which can remove Pb(II) and Cd(II) from aqueous solution via adsorption [17]. Drug delivery requires polyampholyte to exhibit a conformational response to the external stimuli such as pH change, but other applications of polyampholyte rely on its strong interaction, especially adsorption, with substances such as proteins, foulants, metallic ions, and the like. The adsorption of random polyampholyte on a variety of absorbents was studied [17,18,19,20], while the adsorbents studied in the adsorption test of block polyampholyte are mainly silicon substrate [7]. The adsorption of block polyampholyte on other adsorbents, e.g., bentonite (Bent), was rarely reported.

Bent is a special kind of adsorbent carrying both high-density excessive negative charges on its flat layer surface and positive charges at its edges in aqueous solutions. The adsorption of polyampholyte bearing net negative charge on Bent acts as a decisive factor for the properties of water-based drilling fluid, as the adsorbed polyampholyte with excessive negative charges provides Bent with entropic protection against salt intrusion and heating. The adsorption of random polyampholyte on Bent has been reported in a previous paper [21]. The molecular composition and intrinsic viscosity of random polyampholyte exerted different effects on the adsorption of random polyampholyte. However, to the best of our knowledge, the adsorption of block polyampholyte on Bent and similar adsorbents has rarely been discussed, as most of the polymers interacted with Bent studied in previous papers can be classified as the polycation or polyanion based on polyacrylamide [22,23,24].

The main difference between random polyampholyte and block polyampholyte lies in their monomer sequence distribution. To study the effect of monomer sequence, charge asymmetry and molecular weight on the adsorption of polyampholyte on Bent, the authors availed themselves of the reversible addition fragmentation transfer polymerization (RAFT) method to synthesize both random polyampholyte via a one-step reaction and block polyampholyte with net negative charges via a two-step reaction, and then conducted the adsorption experiments to compare the adsorptive ability between them.

## 2. Materials and Methods 

### 2.1. Sample Preparation

Methacrylamido propyl trimethyl ammonium chloride (MAPTAC, 50 wt % in water) was purchased from Sigma Aldrich (Shanghai, China). MAPTAC was purified by using column chromatography and was titrated with 0.1 mol/L AgNO_3_ solution to determine the concentration. 2-acrylamido-2-methyl propane sulfonic acid (AMPS) was provided by Sinopharmacy Corporation (Shanghai, China) and was purified by recrystallization in CH_3_OH twice before use. Azodiisobutyronitrile (AIBN) was purchased from Chengdu Kelong Chemical Corporation (Chengdu, China) and was purified by recrystallization in CH_3_CH_2_OH for three times before use. NaOH, NaCl, CH_3_OH, CH_3_CH_2_OH were provided and used without purification by Chengdu Kelong Chemical Corporation. All the chemicals above are of analytic reagent. The Bent used in this paper was purchased from Xinjiang Xiazijie Company (Urumqi, China). The bentonite was purified via a following procedure: Five weight percent Bent and 0.25 wt % Na_2_CO_3_ were dissolved in deionized water, and the solution was aged at room temperature for 24 h; the mixture was centrifugated at 10,000 RPM to isolate Bent; the Bent was dried under 110 °C, and then sifted with a mesh #100. The purified Bent was tested according to the China Industrial Standard GB/T20973-2007, the cation exchange capacity (CEC) was 85.8 cmol·kg^−1^, and the swelling volume was 38.5 mL·g^−1^.

**Figure 1:**
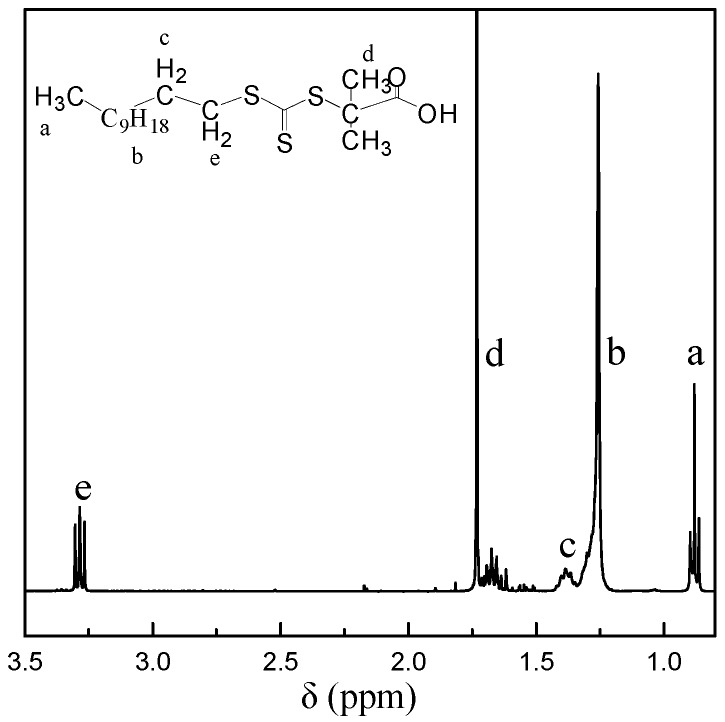
The ^1^H NMR spectrum of 2-[Dodecylthio(thiocarbonyl)thio]-2-methylpropionic acid (DDMAT).

The synthesis of 2-[Dodecylthio(thiocarbonyl)thio]-2-methylpropionic acid (DDMAT) was conducted according to a previous related paper [25], and the molecular structure of DDMAT was characterized by ^1^H NMR, as shown in Figure 1.

**Figure 2:**
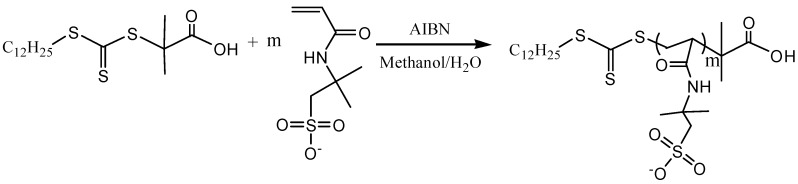
The synthesis route of *Macro*-PAMPS (first step).

**Figure 3:**
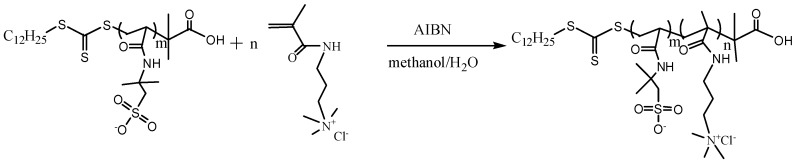
The synthesis route of PAMPS-b-PMAPTAC (second step).

The block polyampholyte PAMPS-b-PMAPTAC (P*block*) was produced via a two-step reaction. This first step was to synthesize a macro-chain transfer agent, *Macro*-PAMPS (Figure 2), and the second step was to synthesize P*block* via a RAFT polymerization between *Macro*-PAMPS and MAPTAC monomers (Figure 3).

*Macro*-PAMPS was synthesized according to the following procedure: CH_3_OH and deionized water (3:1 in volume ratio) were added into a flask in icy water, and the solution was purged with Ar for 30 min. AMPS monomers were added to the solution, and then NaOH was used to adjust the pH of the solution to around neutral. The flask was distilled to vacuum, and was purged with Ar for 10 min, which was repeated for three times. DDMAT and azobisisobutyronitrile (AIBN), dissolved in CH_3_OH/H_2_O, were successively added to the solution via injection. The flask was sealed and heated to 65 °C, and the reaction lasted for 7 h. When the polymerization was completed, the product was transferred to a dialysis bag (3500 D) in deionized water for 24 h. The dialysis treatment was repeated for three times. Then the product was purified using the freeze-drying technique.

The preparation of P*block* was specified as follows: a certain amount of PAMPS-DDMAT was dissolved in CH_3_OH and deionized water (3:1 in volume ratio), and MAPTAC was added later. After all materials were dissolved, the flask containing the solution was distilled to vacuum and injected with Ar for 10 min, which was repeated for three times. AIBN solution was added to the solution via injection. The flask was sealed and was heated to 65 °C. After reacting for 6 h, the product in the flask was distilled with CH_3_CH_2_OH four times. Then the product was transferred to the vacuum oven and dried at 50 °C for 24 h.

The random polyampholyte PAMPS-*s*-PMAPTAC (P*random*) was prepared using one-step reaction. AMPS monomers and MAPTAC monomers were dissolved in the mixture of CH_3_OH and deionized water (3:1 in volume ratio). After removal of dissolved O_2_ in the solution by injecting Ar for 10 min, another solution containing both AIBN and DDMAT was injected into the monomer solution. The following procedure was the same as that of P*block*.

### 2.2. FTIR

The infrared spectra of polymers were tested using a WQF-520 FT-IR device (scans: 16, resolution: 0.5 cm^−1^) from Beijing Rayleigh Analytical Instrument Corporation, Beijing, China.

### 2.3. ^1^H NMR

The ^1^H NMR spectra were obtained using a Bruker Ascend^TM^ 400 MHz NMR spectrometer (D_2_O as solvent, narrow bore, static, relaxation time was 2 s, Bruker Company, Billerica, MA, USA).

### 2.4. GPC

The molecular weight distribution of polymers was tested with Waters e2695 GPC (solvent: 1 mol/L NaNO_3_, 0.5 mL/min, 35 °C, polyacrylamide of narrow distribution as the standard substance, Waters Company, Milford, MA, USA).

### 2.5. SEM

The microstructure of polymers in aqueous solutions was observed using an FEI QUANTA450 (Thermo Fisher Scientific, Hillsboro, OR, USA) scanning electron microscope (SEM), with a pretreatment including quick-freeze of the solutions, sublimation of H_2_O, and gold spraying in the surface of the samples.

### 2.6. Zeta Potential

The zeta potential of polymer solutions was tested using a Brookhaven ZetaPALS instrument (Brookhaven Instrument Corporation, Long Island, NY, USA). Five replications were conducted in each zeta potential test.

### 2.7. XRD

The X-ray diffraction (XRD) tests were performed using X’PERT PRO MPD X-ray diffractometer (Malvern Panalytical, Almelo, The Netherlands), operated at 40 kV and150 mA with Cu-Kα radiation (λ = 0.154056 nm) at a scanning speed of 0.02 °/s from 3° to 20°.

### 2.8. Adsorption Tests

The adsorption test was carried out as follows: First, 5 wt % purified Bent was dissolved in deionized water, and the solution was stirred for 24 h under a certain temperature; 5 wt % polymer solution was prepared in the same way. Then the two solutions were mixed together with a volume ratio of 2:1, and NaCl of 0 to 30 wt % was added. Third, the mixture was stirred for 0.1 to 24 h under 35 to 65 °C. Forth, the mixture was centrifugated at 10,000 RPM to separate the Bent with adsorbed polymers at the bottom of a centrifuge tube. Fifth, the content of polymers in Bent was calculated based on the content of carbon in Bent, using an apparatus of element analysis, Elementar Vario EL-III (Elementar Company, Langenselbold, Hesse, Germany). The content of carbon in purified Bent was tested and the result was 0 wt %.

## 3. Results and Discussion

### 3.1. Characterization

**Figure 4:**
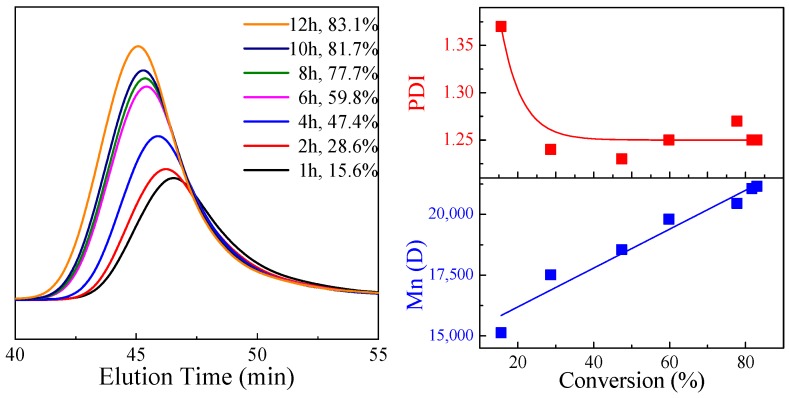
The Gel Permeation Chromatography (GPC) data of *Macro*-PAMPS prepared in the first step.

The molecular weight of synthesized *Macro*-PAMPS in the first step (Figure 2), was characterized by GPC, as shown in Figure 4.

The conversion rate of AMPS monomers increased as the reaction elongated, accompanied by a shift of the eluted peak towards the left, indicating that the molecular weight of the *Macro*-PAMPS increased as the monomer conversion enhanced. Mn and the monomer conversion appeared to be a linear relationship, showing that the chain propagation of AMPS monomers was well controlled in the first step using the RAFT method. The polydispersity index (PDI) stayed at around 1.25 after the conversion increased to over 30%. All the data indicated that *Macro*-PAMPS with a narrow PDI was successfully synthesized.

**Figure 5:**
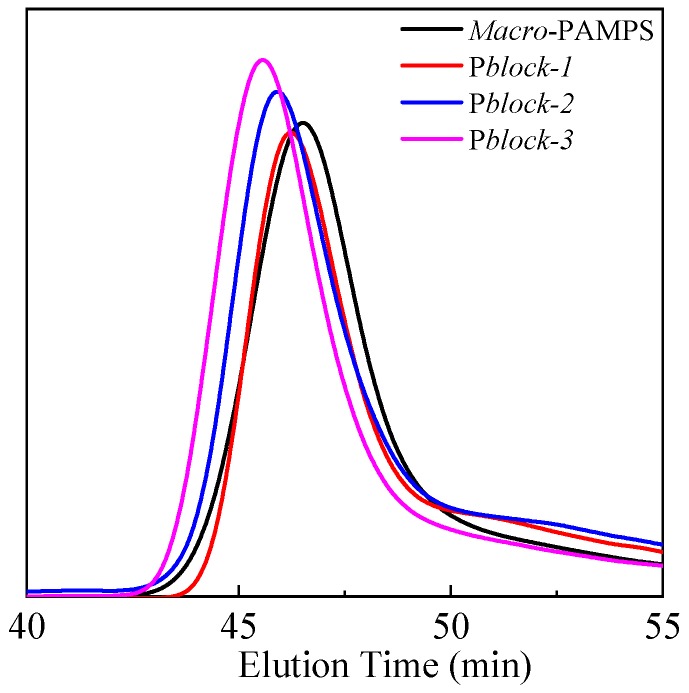
The elution peaks of *Macro*-PAMPS prepared in the first step and block polyampholyte synthesized in the second step.

The products of the second step, P*block*, were also characterized using GPC, as shown in Table 1 and Figure 5. As the addition of MAPTAC monomers increased, the ratio of MAPTAC units to AMPS units in P*block* increased along with a growing molecular weight of P*block*, supported by the shift of elution peaks towards the left (Figure 5). PDI of P*block* also grew larger but was still under control and less than 1.5.

The PDI of P*block* and that of *Macro*-PAMPS were almost the same, demonstrating that block polyampholyte with narrow molecular weight distribution was successfully prepared.

**Figure 6:**
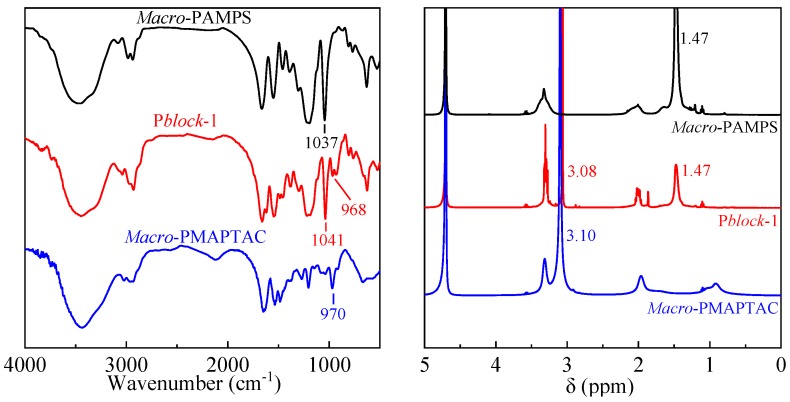
FTIR and ^1^H NMR of *Macro*-PAMPS, P*block*-1, *Macro*-PMAPTAC.

Next, the molecular structure of polymers was characterized by FTIR and ^1^H NMR (Figure 6).

The band around 1041 cm^−1^ corresponded to the symmetric stretching vibration of –SO^3−^ in AMPS unit, and the signal around 970 cm^−1^ was attributed to the stretching vibration of quaternary ammonium group in MAPTAC moieties. The peak at 1.47 ppm was introduced by the –CH_3_ in AMPS unit, while the peak around 3.10 ppm corresponded to the quaternary ammonium group in MAPTAC moieties. The results demonstrated that the target P*block* have been successfully synthesized via two-step reaction using the RAFT method.

A batch of polymers carrying net negative charges including *Macro-*PAMPS, block polyampholyte and random polyampholyte, as shown in Table 2, were synthesized using the RAFT methods for the adsorption test by tuning the feed ratio of AMPS monomers and MAPTAC monomers.

Considering that quaternary ammonium groups and sulfonate groups are stable in a wide range of pH under room temperature, the polymers in Table 2 have little sensitivity to the pH variation of the solutions. As the following adsorption tests were carried out at neutral pH, the precipitation of both random polyampholyte and block polyampholyte cannot be observed as their isoelectric point (IEP) point region exists at pH < 2. 

### 3.2. The Microstructure of Polyampholyte Solutions

**Figure 7:**
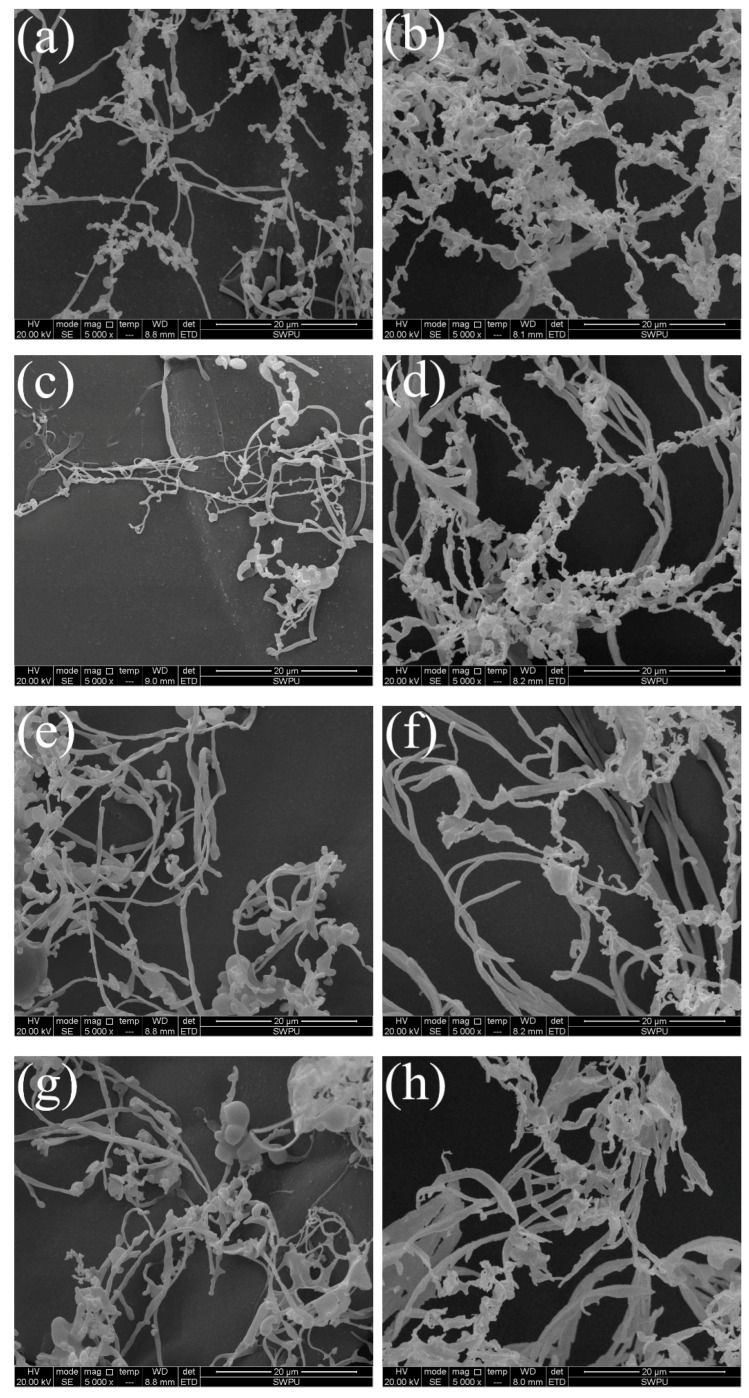

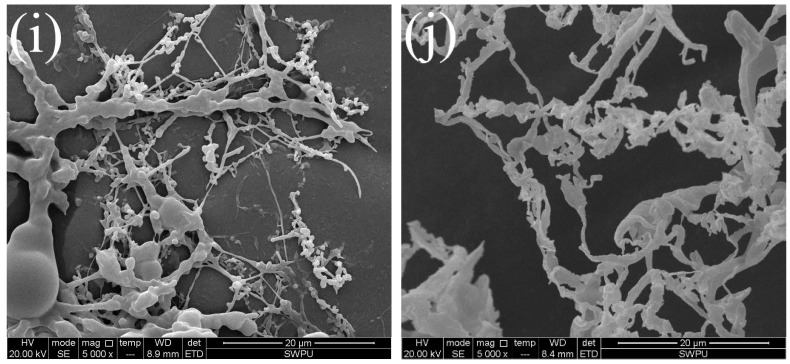
The microstructure of *Macro*-PAMPS, P*block*-4, P*block*-5, P*block*-6, and P*random* in the absence (**a**,**c**,**e**,**g**,**i**) and presence (**b**,**d**,**f**,**h**,**j**) of NaCl.

The microstructure of polymers (Table 2) before (0.05 wt % polymer) and after (0.05 wt % polymer +0.05 wt % NaCl) the intrusion of salt was studied using SEM, and the results are shown as Figure 7.

The chains of P*random* extended upon the intrusion of salt. In deionized water, the chains of random polyampholyte were inclined to be in globular conformation, driven by the intensive electrostatic attraction between adjacent oppositely charged groups. The intrusion of Na^+^ along with the solvent into the space among the entangled chains resulted in a screening of the electrostatic attraction. Therefore, the condensed chains tended to expand in a disordered way.

Both P*block* samples and *Macro*-PAMPS suffered less from the addition of NaCl. The oppositely charged groups of P*block* samples were partially distributed along the chain, and their electrostatic interaction exerted less effect on the microstructure compared with that of P*block*.

The zeta potential of polymers (Table 3) in the presence and absence of NaCl was in agreement with the molecular parameters of these samples. Macro-PAMPS had the lowest zeta potential. As more MAPTAC units were introduced into P*block*, the zeta potential began to increase. The molecular weight also influenced the zeta potential, as Pblock-6 had a lower zeta potential than Pblock-5. After the addition of salt, the zeta potential of all samples suffered greatly, as Na^+^ considerably compressed the hydration layer of charged groups.

### 3.3. The Adsorptive Behavior of Polyampholytes

**Figure 8:**
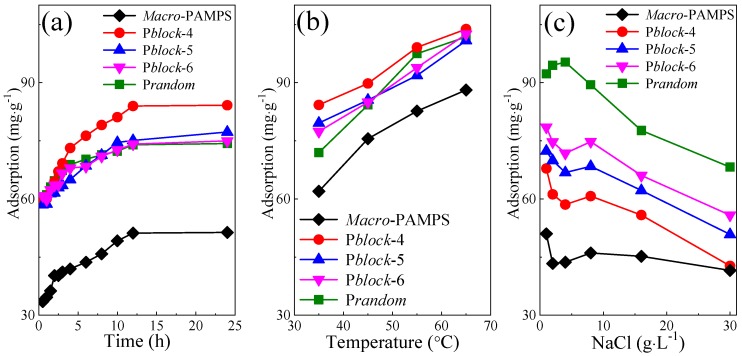
The effect of time (**a**), temperature (**b**), salt (**c**) on the adsorption of *Macro*-PAMPS, block polyampholyte and random polyampholyte.

The adsorption tests were conducted in aqueous solutions, with stirring time, temperature, and salinity as the external factors, as shown in Figure 8.

The experiments on adsorption kinetics were carried out in deionized water at 30 °C. In Figure 8a, it seemed that no distinct difference among the adsorption of block polyampholyte and that of random polyampholyte, except that P*block*-4 exhibited a stronger affinity to Bent. *Macro*-PAMPS had much lower adsorption than polyampholyte, as the former only carries polar groups and anionic groups which had lower adsorptive ability than cationic groups. Several points should be emphasized here. The first point is that even an intense interaction between cationic groups and anionic groups in random polyampholyte and block polyampholyte existed according to the observed microstructure (Figure 7), cationic groups in the chains still provided the polyampholyte with more potential adsorption sites than polar groups did in the case of polyanion. The second point is that even if the adsorbent and the adsorbate carry the same type of charge, adsorption can still occur between them. The third point is that the adsorption equilibrium of all polymers in the adsorption experiments was reached after 12 h.

The influence of heating on the adsorption (the Bent-polymer solution was mixed for 12 h) of polymers was also studied in deionized water. The adsorption of block polyampholyte and random polyampholyte was almost the same, according to Figure 8b. An interesting phenomenon was observed that the adsorption of *Macro*-PAMPS and polyampholyte were promoted by the increase of temperature in the range of 35 to 65 °C. The first possible explanation is that the pronounced thermal motion induced by increased temperature weakened the electrostatic binding between oppositely charged groups, and cationic groups getting rid of the electrostatic bound adsorbed on Bent’s negatively charged layer surface. The second explanation is that a partial expansion of the macromolecular chains, as a consequence of the enhancement of the solvent quality, may also favor the escape of cationic groups from the entangled or even condensed polymer segments [26].

The adsorption tests (the Bent-polymer solution was mixed for 12 h) in the presence of NaCl were carried out at 30 °C. In Figure 8c, the salt exerted a negative effect on the adsorption of all polymers. As Na^+^ permeated into the space among charged groups and compressed the double electric layer of them, the entangled or even condensed chains via electrostatic attraction tended to stay away from one another. The inter-chain entanglement driven by hydrogen bond also suffered significantly from the intrusion of ions. On the other hand, the double electric layer of Bent was also compressed by ionized NaCl, and a considerable amount of Na^+^ surrounded the interlayer surface of Bent. Therefore, the interactions between Bent and charged groups or polar groups became less effective, leading to less adsorption of polymers.

P*block*-6, sharing a similar monomer ratio with P*block*-5 but a higher Mn than the later, displayed higher adsorption than P*block*-5. This result may reveal that the average length of the chain mainly determines both the number of potential adsorptive groups and the adsorption of the polymer. 

It is also worth mentioning that P*random* suffered less from the increased salinity than P*block* samples. Considering that the former exhibited less adsorption than the later in the absence of NaCl, we can infer that molecular weight contributed more to the adsorption of polyampholyte in deionized water, while the content of cationic group dominated the adsorption of polyampholyte in the presence of salt, as P*random* had a higher percentage of cationic moieties but lower Mn compared with P*block* (Table 2). *Macro*-AMPS still had the lowest adsorption after the intrusion of salt, stemming from its lowest zeta potential (Table 3).

**Figure 9:**
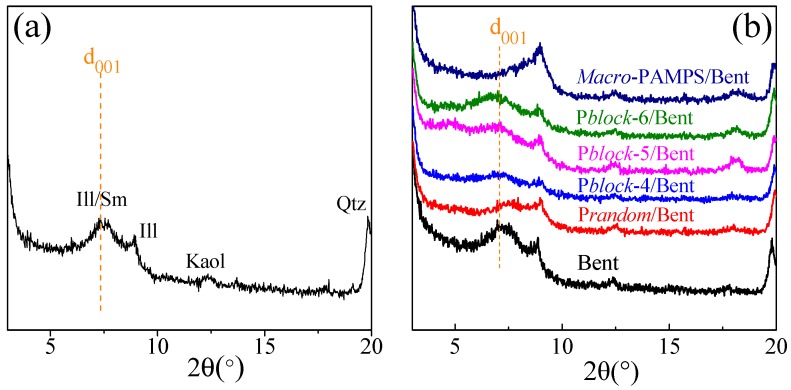
The X-ray diffraction (XRD) analysis of bentonite (Bent) (**a**) and Bent with adsorbed polymers (**b**).

The structure of Bent before and after the adsorption of polymers was characterized by XRD. Figure 9a illustrated the composition of Bent. Ill/Sm, Ill, Kaol and Qtz refer to Illite/Smectite, Illite, Kaolinte and Quartz, respectively. Polymers mainly adsorbed on Illite/Smectite. According to Figure 9b, the d_001_ basal spacing (2θ ≈ 7°) of Bent was weakened and moved towards a small degree as different polymers adsorbed in flat layer surface [27]. Assuming pole conformation, fence conformation or pancake conformation, the polymer chains enclosed Bent plates, and increased both the electrostatic repulsion and entropic repulsion among those particles, resulting in a disordered distribution of Bent lattice planes. More importantly, the interlayer spacing of Bent lattice planes grew larger as polymers intercalated into the interlayer space in Bent particles, as the d_001_ basal spacing of P*block*-4/Bent, P*block*-5/Bent, and P*block*-6/Bent moved towards a smaller angle compared with purified Bent. This result indicated that the polymers adsorbed on the flat layer surface of Bent particles. The cationic groups in P*block* were distributed as a segment, interacting with the negatively charged interlayer surface of Bent via a multi-point adsorption.

The d_001_ basal spacing of *Macro*-PAMPS/Bent and P*random*/Bent had rarely shifted, which means that the adsorption of these polymers exhibited less effect on the expansion of Bent interlayer space. The statistical distribution of positive charged groups in P*random* had an adverse effect on the interaction between these cationic groups and the negatively charged interlayer surface of Bent, resulting in both lower d_001_ basal spacing of P*random*/Bent and less adsorption.

As block polyampholyte bearing net negative charges exhibited a greater adsorptive ability on Bent than random polyampholyte according to the analysis of XRD tests and adsorption tests, the former may find a broad application in water-based mud of oil and gas industry and the synthesis of polymer-clay nanocomposites.

## 4. Conclusions

In this paper, we synthesized block polyampholyte using the RAFT method, with AMPS and MAPTAC monomers. A macro transfer agent *Macro*-PAMPS was prepared, and then the copolymerization between *Macro*-PAMPS and MAPTAC monomers initiated by AIBN was conducted. FTIR, ^1^H NMR, and GPC results demonstrated that the target block polyampholyte was successfully synthesized. 

The partial distribution of groups carrying like charges in polyampholyte led to an intense entanglement of polymer chains, while the random distribution of oppositely charged groups resulted in a condense conformation of the chains. Addition of salt weakened the electrostatic interaction between charged groups and, therefore, influenced the microstructure of polyampholyte. The zeta potential of *Macro*-PAMPS and polyampholyte decreased considerably upon the intrusion of NaCl.

The adsorption kinetic study revealed that after 12 h, *Macro*-PAMPS and polyampholyte reached the adsorption equilibrium on Bent. Increasing temperature in a proper range contributed to the adsorption while the introduction of NaCl affected the adsorption. In deionized water, a better way to increase the adsorption of polyampholyte on Bent is to increase its molecular weight; in the presence of salt, introducing more cationic moieties seems to be a more practical method to enhance the adsorption of polyampholyte.

Block polyampholyte had a stronger adsorptive affinity to Bent than random polyampholyte in the absence of salt, and the former showed higher intercalation ability in enlarging the d_001_ basal spacing of Bent.

## Figures and Tables

**Table 1:** The molecular parameters of *Macro*-PAMPS prepared in the first step and block polyampholyte synthesized in the second step.

Sample	n_AMPS_:n_MAPTAC_	Mn (D)	PDI
*Macro*-PAMPS	-	10,500	1.21
P*block*-1	4.35:1	12,600	1.25
P*block*-2	2.13:1	14,300	1.28
P*block*-3	0.85:1	16,600	1.35

**Table 2:** The molecular parameters of polymer samples used in the following adsorption tests.

Sample	n_AMPS_:n_MAPTAC_	Mn (D)	PDI
*Macro*-PAMPS	-	28,600	1.23
P*block*-4	5.99:1	24,500	1.30
P*block*-5	3.99:1	24,700	1.39
P*block*-6	4.01:1	26,700	1.35
P*random*	3.56:1	21,800	1.41

**Table 3:** The zeta potential of polymer solutions.

Sample	Deionized Water	Brine Water
*Macro*-PAMPS	−93.44	−49.14
P*block*-4	−78.57	−36.94
P*block*-5	−40.68	−13.48
P*block*-6	−50.59	−18.68
P*random*	−38.30	−12.43
